# Crystal structure and Hirshfeld surface analysis of 4-bromo-6-phenyl-6,7-di­hydro-5*H*-furo[2,3-*f*]isoindol-5-one

**DOI:** 10.1107/S2056989025007170

**Published:** 2025-08-15

**Authors:** Kseniia A. Alekseeva, Mikhail S. Grigoriev, Irina A. Kolesnik, Narmin A. Murshudlu, Khudayar I. Hasanov, Roya Z. Nazarova, Mehmet Akkurt, Gizachew Mulugeta Manahelohe

**Affiliations:** a RUDN University, 6 Miklukho-Maklaya St., Moscow 117198, Russian Federation; b Frumkin Institute of Physical Chemistry and Electrochemistry, Russian Academy of Sciences, Leninsky prosp. 31, bld. 4, 119071, Moscow, Russian Federation; c Institute of Physical Organic Chemistry, National Academy of Sciences of Belarus, 220072 Minsk, Belarus; d Department of Chemistry, Faculty of Natural Sciences, Sumgait State University, Baku Str. 1, AZ 5008, Sumgait, Azerbaijan; e Azerbaijan Medical University, Scientific Research Centre (SRC), A. Kasumzade St. 14, AZ 1022, Baku, Azerbaijan; f Baku Engineering University, Khirdalan, Hasan Aliyev str. 120, AZ0101, Absheron, Azerbaijan; g Department of Physics, Faculty of Sciences, Erciyes University, 38039 Kayseri, Türkiye; h Department of Chemistry, University of Gondar, PO Box 196, Gondar, Ethiopia; University of Neuchâtel, Switzerland

**Keywords:** crystal structure, iso­indole, furo[2,3-*f*]iso­indole, IMDAV reaction, Diels–Alder reaction, Hirshfeld surface analysis

## Abstract

The title mol­ecule, C_16_H_10_BrNO_2_, is essentially planar (r.m.s. deviation = 0.004 Å). In the crystal, mol­ecules are linked by C—H⋯O and C—H⋯Br hydrogen bonds, forming ribbons along the *b*-axis direction. Furthermore, π-π inter­actions cause the mol­ecules to form ribbons along the [1 0 ] and [1 0 10] directions [centroid-to-centroid distances = 3.703 (3), 3.734 (3), 3.703 (3), and 3.734 (3) Å]. According to a Hirshfeld surface analysis, H⋯H (33.8%), O⋯H/H⋯O (15.1%), C⋯H/H⋯C (14.6%), Br⋯H/H⋯Br (13.8%), and C⋯C (11.9%) inter­actions are the main contributors to the crystal packing.

## Abstract 2

The title mol­ecule is essentially planar [r.m.s. deviation = 0.004 Å]. In the crystal, C—H⋯O and C—H⋯Br hydrogen bonds link the mol­ecules, forming ribbons along the *b*-axis direction. π–π inter­actions are also observed.

## 1. Chemical context

Iso­indoles and their partially hydrogenated and/or condensed derivatives are widely occurring heterocycles in nature. This scaffold has significant applications in diverse fields, including medicine, photoactive materials, coordination chemistry, and fine organic synthesis. Their unique structural features allow for the creation of derivatives that exhibit a wide range of biological activities. Consequently, developing novel synthetic methods to overcome existing challenges, as well as reactions that leverage iso­indoles to access functionally valuable compounds, has attracted considerable attention (for recent reviews, see: Speck & Magauer, 2013 ▸; We­intraub & Wang, 2023 ▸; Ou-Ichen *et al.*, 2024 ▸).

Over the past decade, our group has explored the construction of fused iso­indoles *via* the intra­molecular Diels–Alder reaction in vinyl­arenes (the IMDAV reaction) (Zaytsev *et al.*, 2021 ▸, 2023 ▸ and a review, Krishna *et al.*, 2022 ▸) and expanded its synthetic utility through the development of multicomponent one-pot cascade transformations (Voronov *et al.*, 2018 ▸; Alekseeva *et al.*, 2023 ▸, 2024 ▸). In a recent study (Alekseeva *et al.*, 2020 ▸), it was demonstrated that 3-(2-fur­yl)allyl­amines and bromo­maleic anhydride react *via* an IMDAV reaction followed by de­hydro­bromination. Furthermore, *in situ*-generated HBr was found to induce an aromaticity transfer from the furan ring to the cyclo­hexane moiety. Based on this observation, we aimed to investigate whether entirely de­hydrogenated fused iso­indole could be synthesized directly from 3-aryl­allyl­amine and halogen-substituted maleic anhydride. Given that the IMDAV reaction between 3-(2-fur­yl)allyl­amines and maleic anhydride yields 5-oxo-4a,5,6,7,7a,8-hexa­hydro-4*H*-furo[2,3-*f*]iso­indole-4-carb­oxy­lic acids (Apponyi *et al.*, 2002 ▸; Deng *et al.*, 2019 ▸), we hypothesized that employing di­bromo­maleic anhydride would facilitate the formation of two carbon–carbon double bonds through successive de­hydro­bromination reactions.

**Figure 1:**
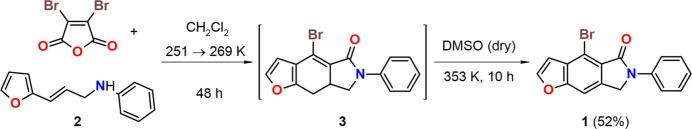
Synthesis of 4-bromo-6-phenyl-6,7-di­hydro-5*H*-furo[2,3-*f*]isoindol-5-one.

Contrary to our expectations, the reaction between *N*-[(2*E*)-3-(furan-2-yl)prop-2-en-1-yl]aniline (**2**) and di­bromo­maleic anhydride was accompanied by simultaneous de­hydro­bromination and deca­rboxylation. The resulting product (**3**) displayed limited solubility in common deuterated solvents. For its characterization by NMR, compound **3** was dissolved in DMSO-*d*_6_ and heated to 353 K to obtain a clear solution. Surprisingly, NMR analysis indicated that DMSO favours oxidation of **3** to yield 4-bromo-6-phenyl-6,7-di­hydro-5*H*-furo[2,3-*f*]isoindol-5-one (**1**) (Fig. 1 ▸). This aromatization reaction was subsequently confirmed using non-deuterated DMSO.
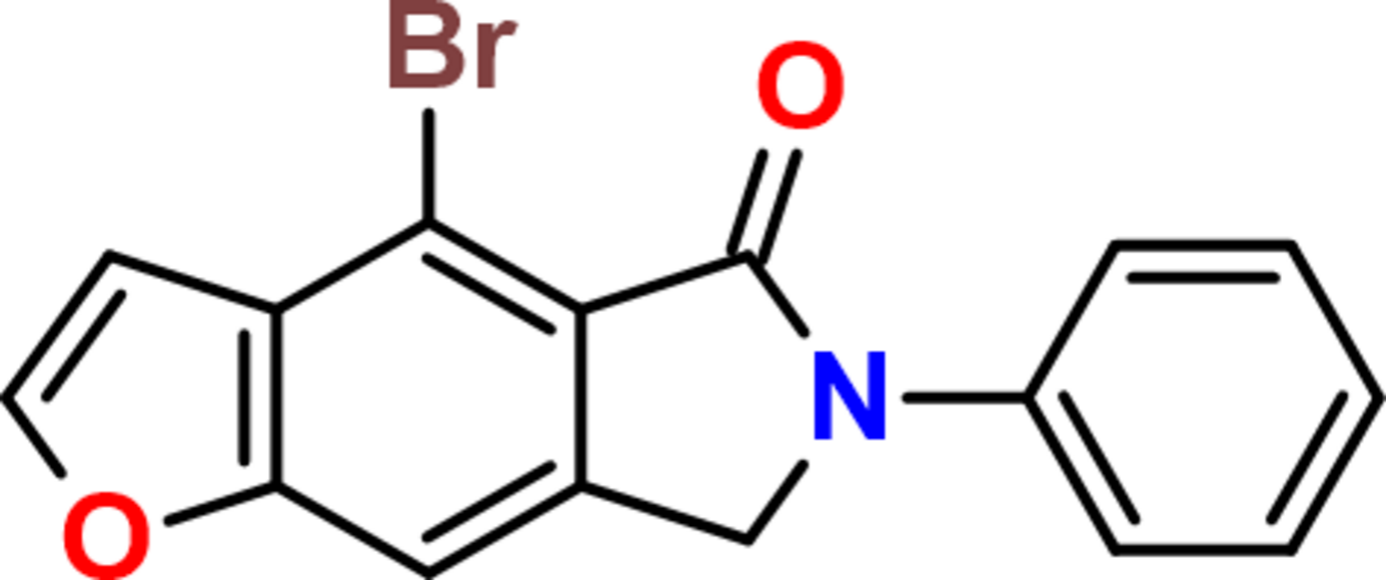


## 2. Structural commentary

**Figure 2:**
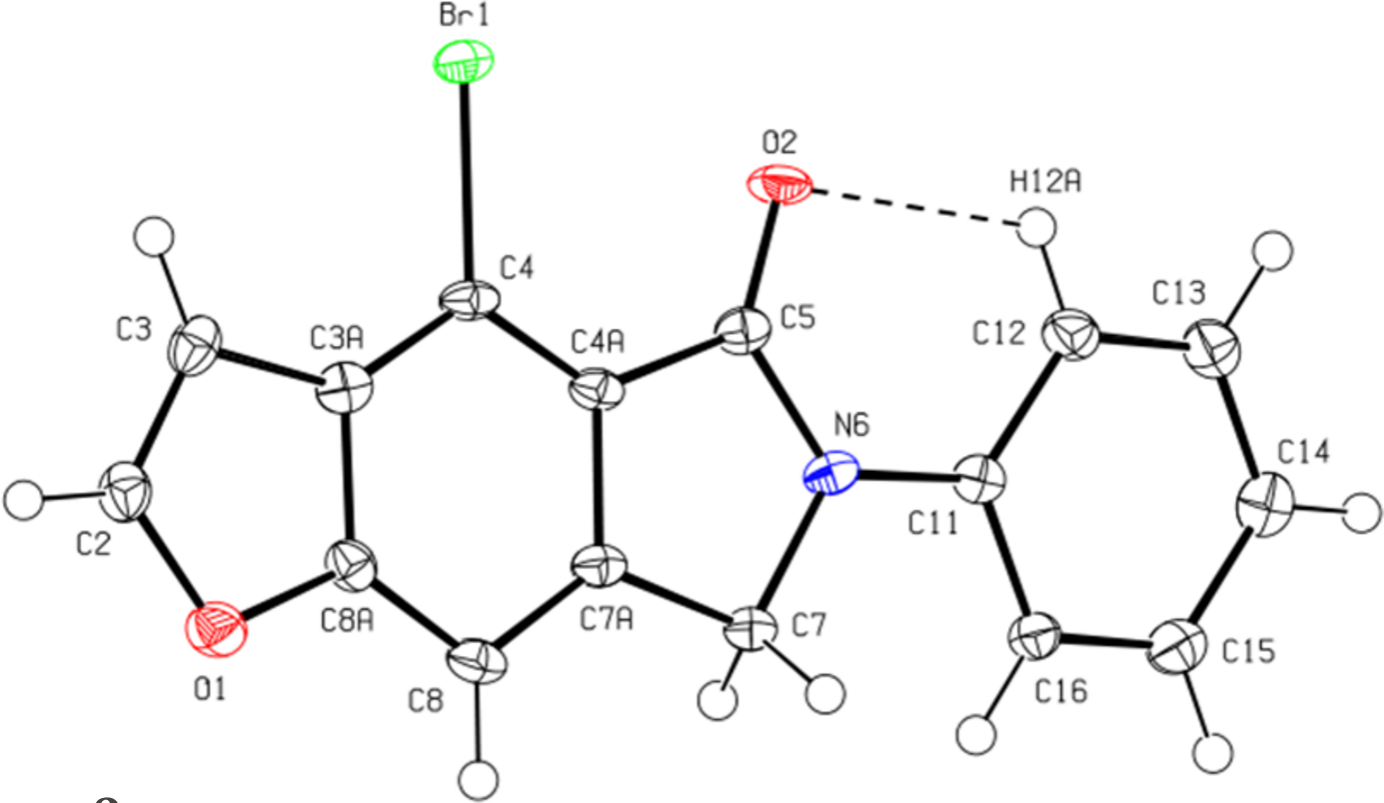
The title mol­ecules showing the atom-labelling scheme with displacement ellipsoids drawn at the 50% probability level.

The conformation of the mol­ecule is stabilized by an intra­molecular C—H⋯O hydrogen bond (Table 1 ▸, Fig. 2 ▸) that forms an *S*(6) motif (Bernstein *et al.*, 1995 ▸). Thus, the mol­ecule is planar except for some hydrogen atoms. The distances of the furthest atoms from the least squares plane of the mol­ecule are −0.153 (4), 0.141 (4), −0.097 (4), and 0.090 (5) Å for atoms C15, C12, C16, and C13, respectively. The C5—N6—C11—C12 and C7—N6—C11—C16 torsion angles are 10.9 (6) and 5.7 (5)°, respectively. The geometric parameters of the title compound are normal and consistent with those of related compounds listed in the *Database survey* (Section 4).

## 3. Supra­molecular features

**Figure 3:**
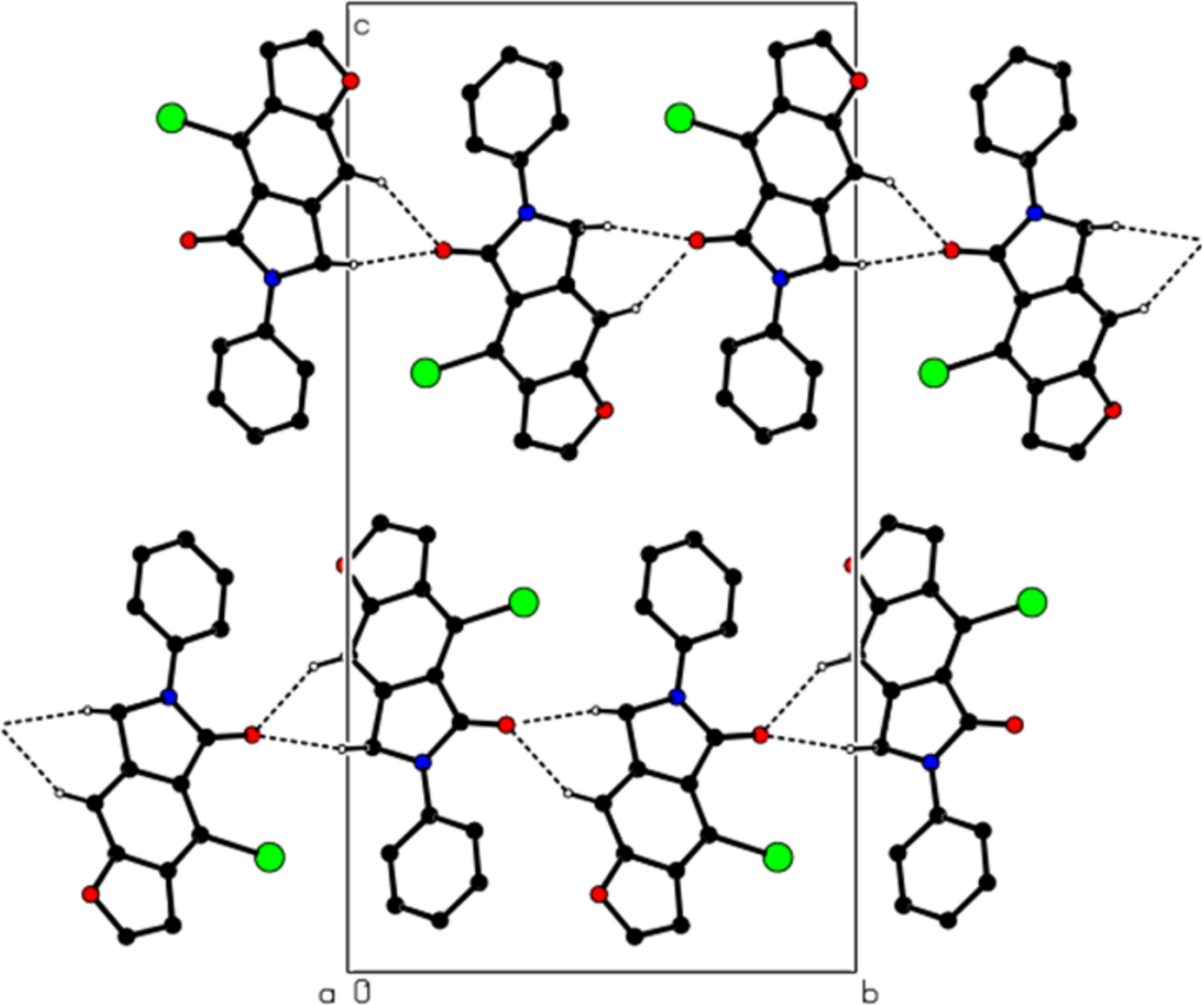
Partial packing of the title compound, viewed down the *a*-axis direction, showing C—H⋯O and C—H⋯Br hydrogen bonds as dashed lines. H atoms not involved in these inter­actions have been omitted for clarity.

**Figure 5:**
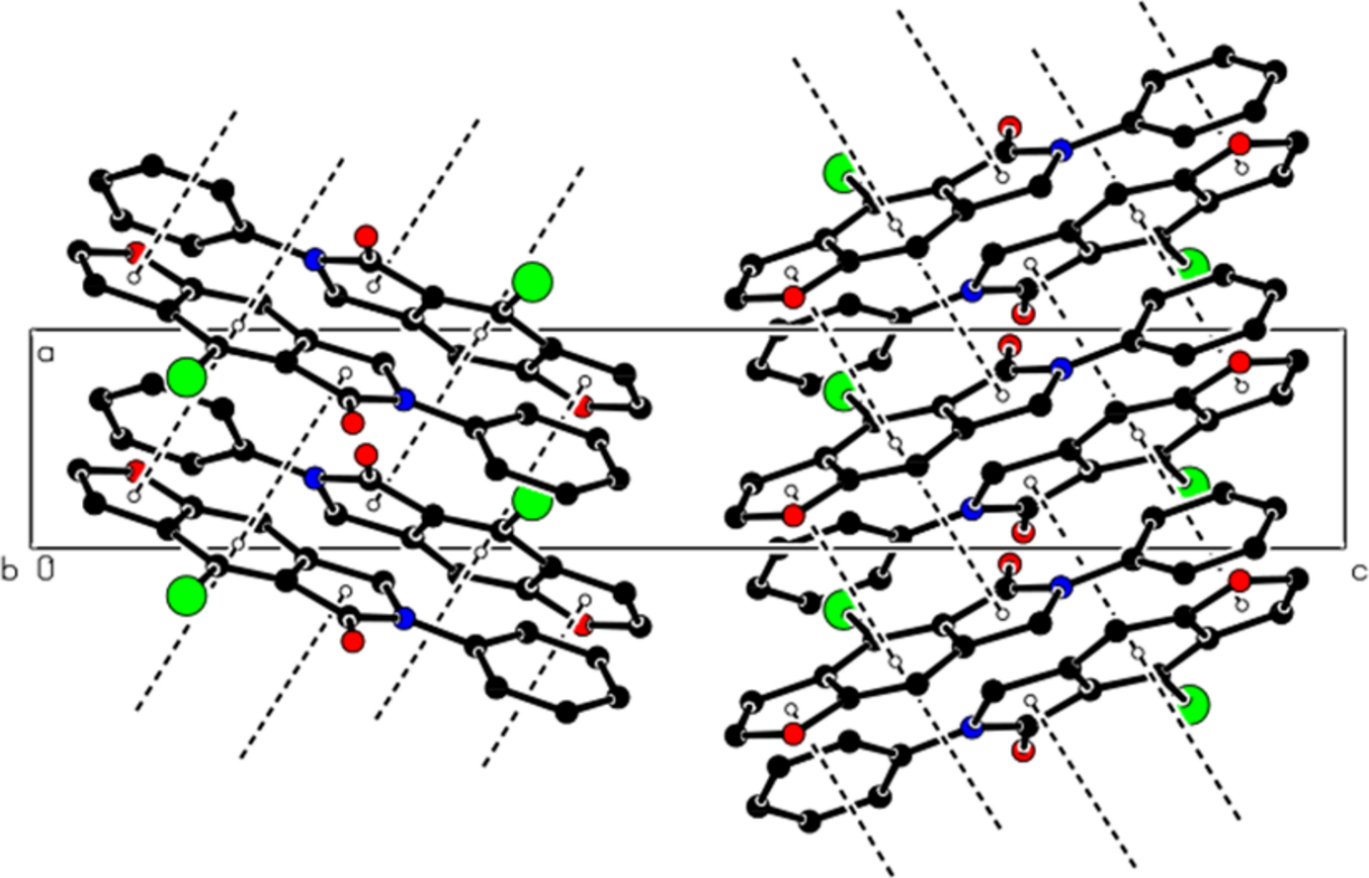
A partial view down the *b*-axis direction showing the π–π inter­actions (dashed lines).

In the crystal, mol­ecules are linked by C—H⋯O and C—H⋯Br hydrogen bonds, forming ribbons along the *b*-axis direction (Table 1 ▸, Figs. 3 ▸ and 4 ▸). The mol­ecules are further linked by π–π inter­actions [*Cg*1⋯*Cg*3^i^ = 3.703 (3) Å, slippage = 1.236 Å; *Cg*2⋯*Cg*3^ii^ = 3.734 (3) Å, slippage = 1.243 Å; *Cg*3⋯*Cg*1^*b*^ = 3.703 (3) Å, slippage = 1.227 Å; symmetry codes: (i) −1 + *x*, *y*, *z*; (ii) 1 + *x*, *y*, *z*; where *Cg*1, *Cg*2 and *Cg*3 are the centroids of the O1/C2/C3/C3*A*/C8*A*, N6/C5/C4*A*/C7*A*/C7 and C3*A*/C4/C4*A*/C7*A*/C8/C8*A* rings, respectively], thus forming ribbons along the [1 0 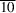] and [1 0 10] directions (Table 1 ▸, Fig. 5 ▸). C—H⋯π inter­actions were not observed.

**Figure 6:**
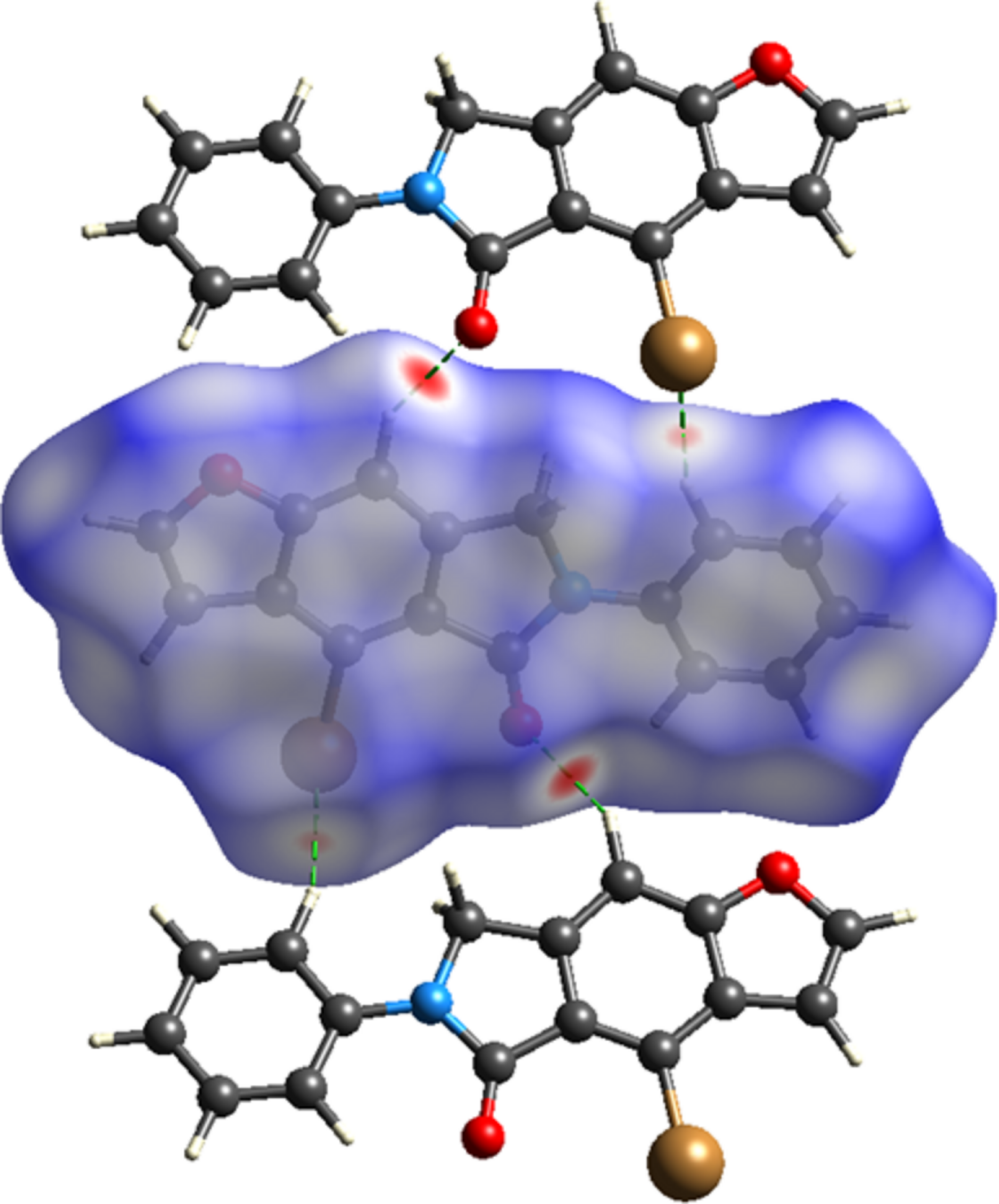
View of the three-dimensional Hirshfeld surface of the title compound plotted over *d*_norm_.

**Figure 7:**
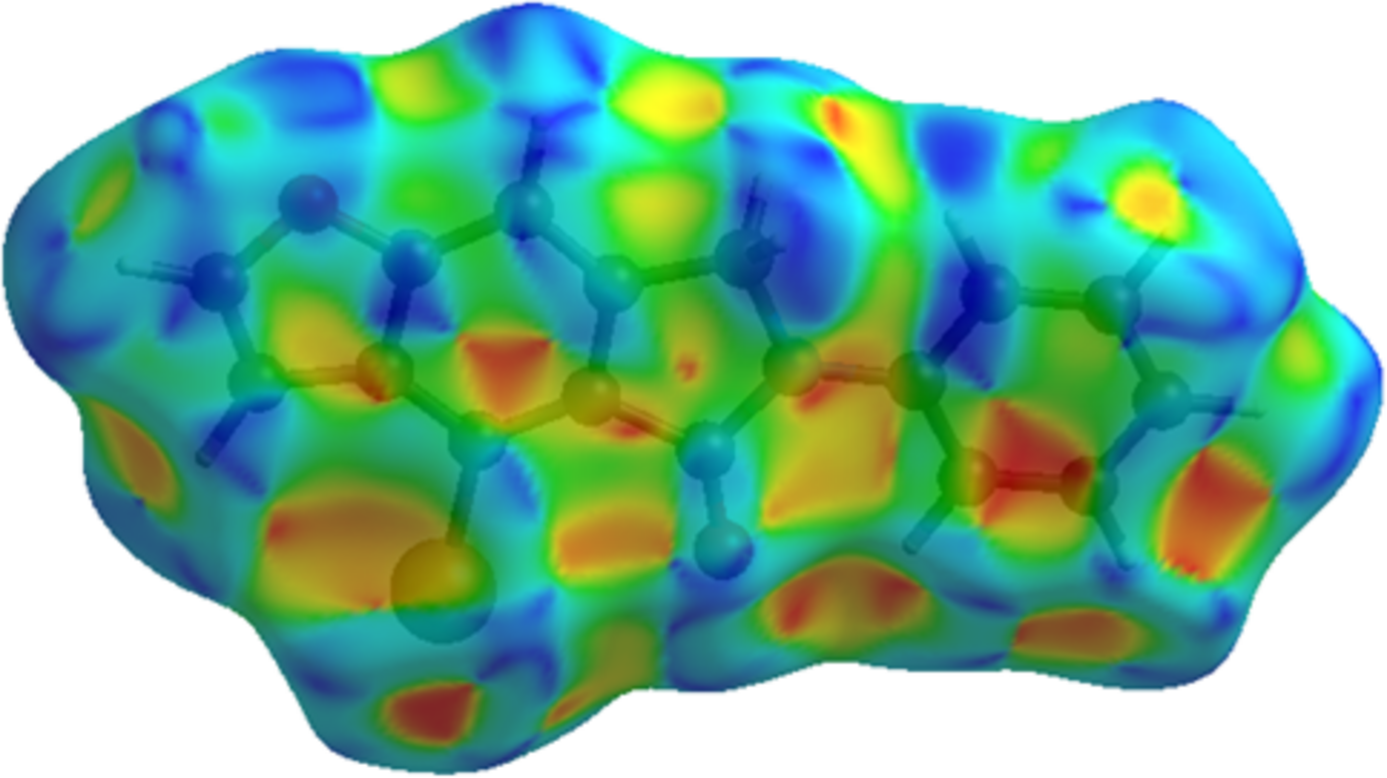
Hirshfeld surface of the title compound plotted over shape-index.

**Figure 8:**
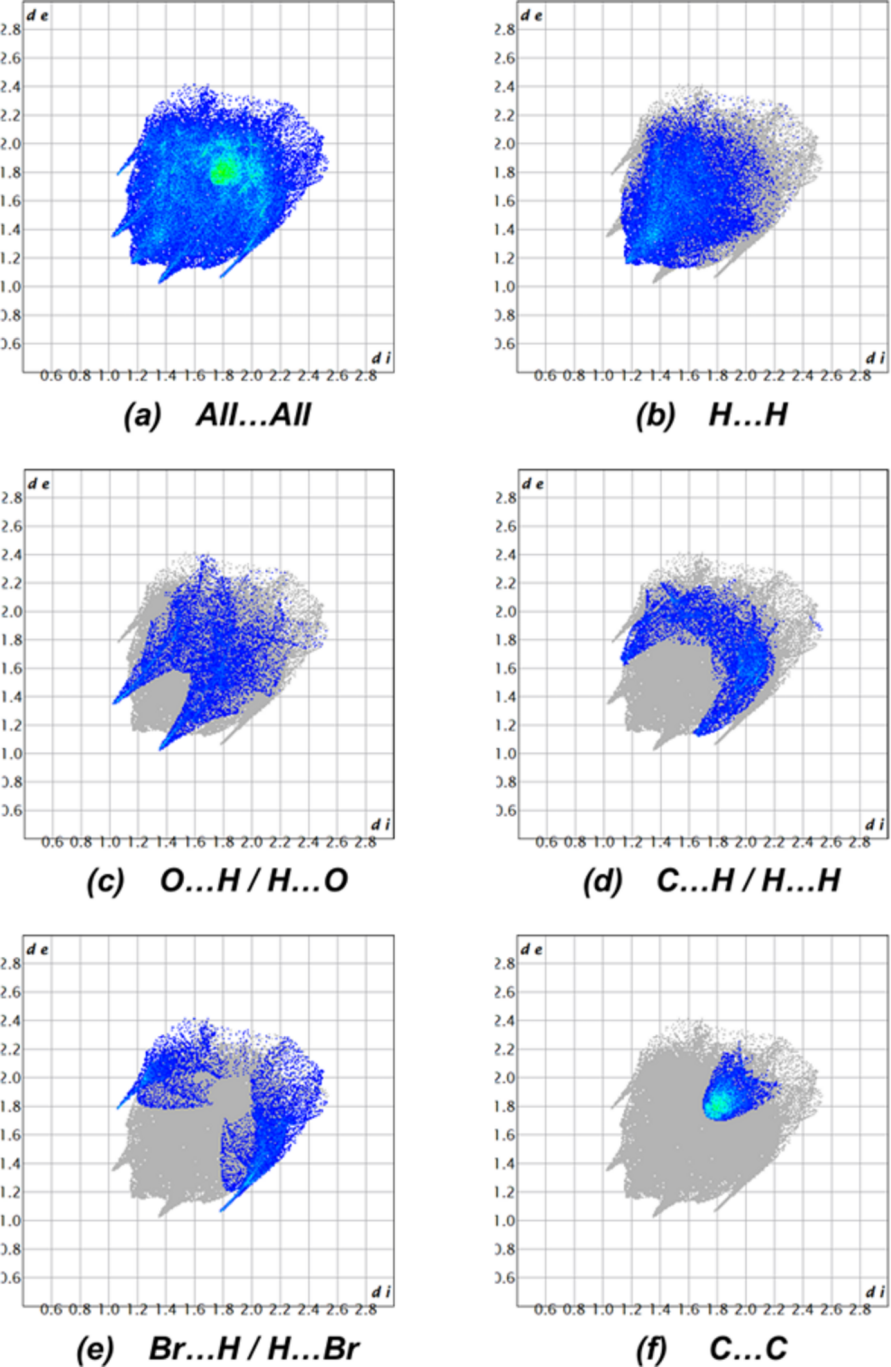
Two-dimensional fingerprint plots, showing (*a*) all inter­actions, and delineated into (*b*) H⋯H, (*c*) O⋯H/H⋯O, (*d*) C⋯H/H⋯C, (*e*) C⋯Br/Br⋯C and (*f*) C⋯C inter­actions [*d*_e_ and *d*_i_ represent the distances from a point on the Hirshfeld surface to the nearest atoms outside (external) and inside (inter­nal) the surface, respectively].

Using *CrystalExplorer 17.5* (Spackman *et al.*, 2021 ▸), a Hirshfeld surface analysis was performed to visualize the inter­molecular inter­actions (Tables 1 ▸ and 2 ▸). The red and blue areas in the Hirshfeld surface plotted over the *d*_norm_ (Fig. 6 ▸) show contacts that are shorter or longer, respectively, than the van der Waals radii, while the white surface shows contacts with distances equal to the sum of the van der Waals radii (Venkatesan *et al.*, 2016 ▸). Significant π–π inter­actions are shown by the Hirshfeld surface’s shape-index (Fig. 7 ▸). Fig. 8 ▸ shows the overall two-dimensional fingerprint plot, and Fig. 8 ▸*b*–*f* shows those delineated into H⋯H (33.8%), O⋯H/H⋯O (15.1%), C⋯H/H⋯C (14.6%), Br⋯H/H⋯Br (13.8%) and C⋯C (11.9%) inter­actions. Smaller contributions are made by C⋯O/O⋯C (4.9%), C⋯Br/Br⋯C (2.6%), N⋯H/H⋯N (1.8%), C⋯N/N⋯C (0.8%), Br⋯Br (0.6%) and O⋯O (0.1%) contacts.

## 4. Database survey

A search of the Cambridge Structural Database (CSD, Version 6.00, last update April 2025; Groom *et al.*, 2016 ▸) gave nine hits including the 4-bromo-6-phenyl-6,7-di­hydro-5*H*-furo[2,3-*f*]isoindol-5-one unit, five of which were closely related to the title compound, *viz.* CSD refcodes HEMVEE (He *et al.*, 2022 ▸), JOGYIP (Zhou *et al.*, 2014 ▸), LESXIS (Horak *et al.*, 2013 ▸), OJIPUV (Zaytsev *et al.*, 2021 ▸) and QADZIH (Zubkov *et al.*, 2016 ▸).

π–π and C—H⋯π inter­actions are observed in the structure of HEMVEE. In JOGYIP, weak C—H⋯O inter­actions lead to the formation of a three-dimensional network and C—H⋯π inter­actions are also observed. In LESXIS, O—H⋯O hydrogen bonds between the carb­oxy­lic and carbonyl groups link alternate independent mol­ecules into chains propagating along the *b*-axis direction. The crystal packing also features weak C—H⋯π inter­actions. In OJIPUV, mol­ecules are connected by C—H⋯O hydrogen bonds, C—H⋯π inter­actions and π–π stacking inter­actions, forming a three-dimensional network. In QADZIH, pairs of O—H⋯O hydrogen bond form dimers with an 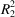
(8) motif. C—H⋯O hydrogen bonds, π–π and C—H⋯π inter­actions were also observed, forming a three-dimensional network.

## 5. Synthesis and crystallization

*N*-[(2*E*)-3-(Furan-2-yl)prop-2-en-1-yl]aniline (1.26 mmol) (**2**) was dissolved in dry CH_2_Cl_2_ (10 mL) and cooled to 251 K. Di­bromo­maleic anhydride (0.32 g, 1.26 mmol) was added, and the mixture was kept at 269 K for 2 d. The resulting precipitate was filtered, dissolved in dry DMSO (10 mL), and stirred at 353 K for 10 h. The mixture was poured into water (50 mL), the resulting precipitate was filtered off, and washed with water (3 × 3 mL). The product was dried in air to constant weight to afford compound **1** as a light-yellow solid (216.6 mg, 0.66 mmol, 52%), m.p. 481–482 K. A single crystal suitable for X-ray analysis was obtained from DMSO-*d*_6_ upon heating to 353 K and slow cooling to r.t.

^1^H NMR (700.2 MHz, DMSO-*d*_6_, 333 K) (*J*, Hz): *δ* 8.21 (*br. d*, *J* = 2.2, 1H, H-2-fur­yl), 7.90–7.89 (*m*, 3H, H-*ortho*-Ph, H-8), 7.45 (*dd*, *J* = 7.6, 2H, H-*meta*-Ph), 7.20 (*dd*, *J* = 7.6, 1H, H-*para*-Ph), 7.11–7.09 (*m*, 1H, H-3-fur­yl), 5.03 (*s*, 2H, H-7) ppm. ^13^C{^1^H} NMR (176.1 MHz, DMSO-*d*_6_, 333 K): *δ* 164.3, 155.7, 148.1, 139.7, 139.2, 129.8, 128.7 (2C), 124.3, 124.0, 119.5 (2C), 110.0, 106.7, 105.7, 48.8 ppm. IR (KBr), *ν* (cm^−1^): 3072, 1686, 1598, 1503, 1452, 1392, 1295, 1266, 1179, 1136, 1035, 878, 754, 690, 606. MS (ESI) *m*/*z*: [*M*]^+^ 327 (Br^79^), 329 (Br^81^).

## 6. Refinement

Crystal data, data collection and structure refinement details are summarized in Table 3 ▸. All hydrogen atoms were positioned geometrically and refined using a riding model, with C—H = 0.95 and 0.99 Å, and with *U*_iso_(H) = 1.2*U*_eq_(C). Five reflections (0 1 1, 0 0 2, 0 1 3, 0 1 2 and 0 2 0) affected by the incident beam-stop, as well as nine reflections showing poor agreement between observed and calculated intensities (3 0 26, 0 13 9, 3 0 25, 4 12 5, −3 8 22, 0 11 8, 4 4 18, 0 5 32 and 0 2 26), were omitted in the final cycles of refinement. The remaining positive and negative residual electron densities are both located near the bromine atom (at 1.11 and 0.77 Å, respectively).

## Supplementary Material

Crystal structure: contains datablock(s) I. DOI: 10.1107/S2056989025007170/tx2102sup1.cif

Structure factors: contains datablock(s) I. DOI: 10.1107/S2056989025007170/tx2102Isup2.hkl

CCDC reference: 2479912

Additional supporting information:  crystallographic information; 3D view; checkCIF report

## Figures and Tables

**Figure 4:**
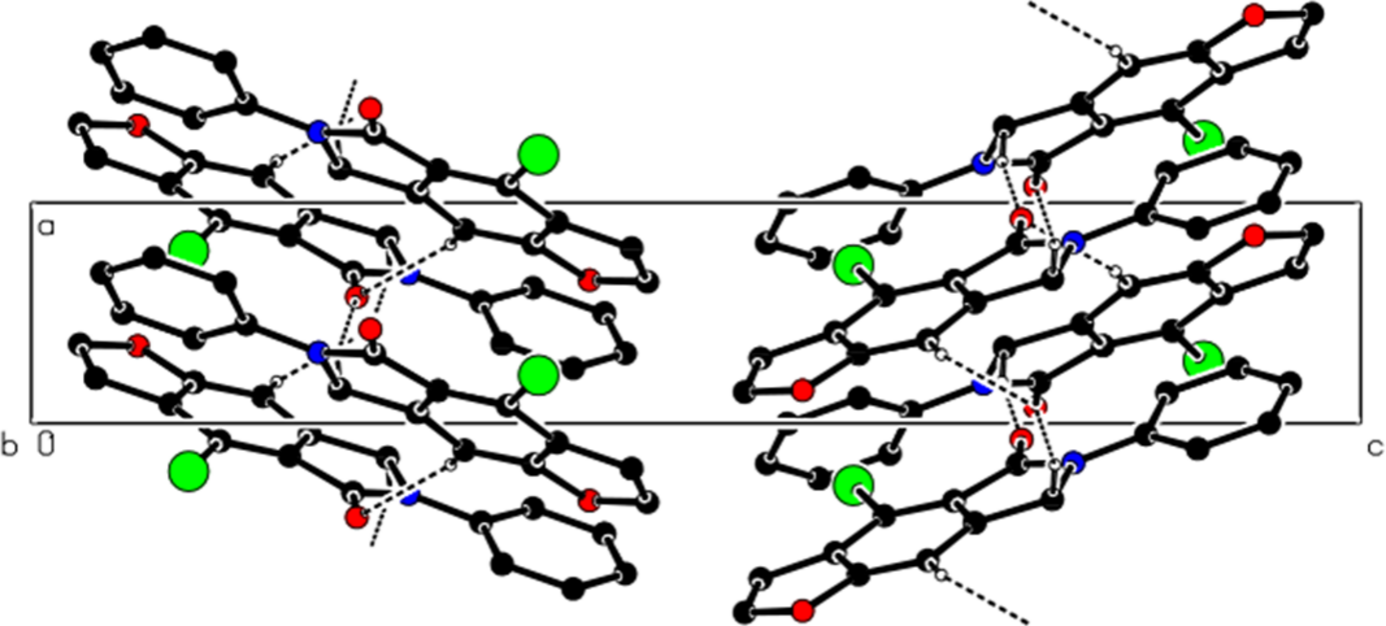
View of the C—H⋯O and C—H⋯Br inter­actions down the *b*-axis direction.

**Table 1:** Hydrogen-bond geometry (Å, °)

*D*—H⋯*A*	*D*—H	H⋯*A*	*D*⋯*A*	*D*—H⋯*A*
C7—H7*A*⋯O2^i^	0.99	2.51	3.463 (5)	162
C8—H8*A*⋯O2^ii^	0.95	2.49	3.348 (5)	149
C12—H12*A*⋯O2	0.95	2.27	2.894 (6)	122
C16—H16*A*⋯Br1^ii^	0.95	2.97	3.864 (4)	157

Symmetry codes: (i) 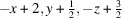; (ii) 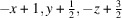.

**Table 2:** Short inter­atomic contacts (Å)

Contact	distance	Symmetry operation
O2⋯H7*A*	2.51	2 − *x*, − 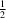 + *y*, 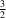 − *z*
O2⋯H8*A*	2.49	1 − *x*, − 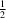 + *y*, 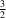 − *z*
H7*B*⋯C11	2.85	−1 + *x*, *y*, *z*
H2*A*⋯H15*A*	2.58	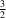 − *x*, 1 − *y*, − 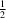 + *z*
H3*A*⋯H3*A*	2.51	− 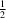 + *x*, 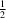 − *y*, 1 − *z*
C14⋯H14*A*	2.91	− 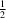 + *x*, 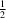 − *y*, 2 − *z*
H2*A*⋯H15*A*	2.54	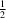 − *x*, 1 − *y*, − 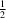 + *z*

**Table 3:** Experimental details

Crystal data	
Chemical formula	C_16_H_10_BrNO_2_
*M*_r_	328.16
Crystal system, space group	Orthorhombic, *P*2_1_2_1_2_1_
Temperature (K)	100
*a*, *b*, *c* (Å)	4.0229 (5), 12.7225 (16), 24.240 (3)
*V* (Å^3^)	1240.6 (3)
*Z*	4
Radiation type	Mo *K*α
μ (mm^−1^)	3.31
Crystal size (mm)	0.32 × 0.30 × 0.04

Data collection	
Diffractometer	Bruker Kappa APEXII area-detector
Absorption correction	Multi-scan (*SADABS*; Krause *et al.*, 2015 ▸)
*T*_min_, *T*_max_	0.417, 0.879
No. of measured, independent and observed [*I* > 2σ(*I*)] reflections	18771, 3594, 2973
*R*_int_	0.077
(sin θ/λ)_max_ (Å^−1^)	0.703

Refinement	
*R*[*F*^2^ > 2σ(*F*^2^)], *wR*(*F*^2^), *S*	0.040, 0.078, 1.02
No. of reflections	3594
No. of parameters	181
H-atom treatment	H-atom parameters constrained
Δρ_max_, Δρ_min_ (e Å^−3^)	0.52, −0.62
Absolute structure	Flack *x* determined using 1016 quotients [(*I*^+^)−(*I*^−^)]/[(*I*^+^)+(*I*^−^)] (Parsons *et al.*, 2013 ▸)
Absolute structure parameter	0.022 (10)

Computer programs: *APEX2* and *SAINT* (Bruker, 2007 ▸), *SHELXS97* (Sheldrick, 2008 ▸), *SHELXL2014* (Sheldrick, 2015 ▸), *ORTEP-3 for Windows* (Farrugia, 2012 ▸) and *PLATON* (Spek, 2020 ▸).

## supplementary crystallographic information

### 4-Bromo-6-phenyl-6,7-dihydro-5H-furo[2,3-f]isoindol-5-one Crystal data

**Table:**

C_16_H_10_BrNO_2_	*D*_x_ = 1.757 Mg m^−^^3^
*M**_r_* = 328.16	Mo *K*α radiation, λ = 0.71073 Å
Orthorhombic, *P*2_1_2_1_2_1_	Cell parameters from 3489 reflections
*a* = 4.0229 (5) Å	θ = 3.0–25.4°
*b* = 12.7225 (16) Å	µ = 3.31 mm^−^^1^
*c* = 24.240 (3) Å	*T* = 100 K
*V* = 1240.6 (3) Å^3^	Plate, colourless
*Z* = 4	0.32 × 0.30 × 0.04 mm
*F*(000) = 656	

### 4-Bromo-6-phenyl-6,7-dihydro-5H-furo[2,3-f]isoindol-5-one Data collection

**Table:**

Bruker Kappa APEXII area-detector diffractometer	2973 reflections with *I* > 2σ(*I*)
φ and ω scans	*R*_int_ = 0.077
Absorption correction: multi-scan (SADABS; Krause *et al.*, 2015)	θ_max_ = 30.0°, θ_min_ = 3.3°
*T*_min_ = 0.417, *T*_max_ = 0.879	*h* = −5→5
18771 measured reflections	*k* = −16→17
3594 independent reflections	*l* = −34→33

### 4-Bromo-6-phenyl-6,7-dihydro-5H-furo[2,3-f]isoindol-5-one Refinement

**Table:**

Refinement on *F*^2^	Hydrogen site location: inferred from neighbouring sites
Least-squares matrix: full	H-atom parameters constrained
*R*[*F*^2^ > 2σ(*F*^2^)] = 0.040	*w* = 1/[σ^2^(*F*_o_^2^) + (0.0274*P*)^2^] where *P* = (*F*_o_^2^ + 2*F*_c_^2^)/3
*wR*(*F*^2^) = 0.078	(Δ/σ)_max_ = 0.001
*S* = 1.02	Δρ_max_ = 0.52 e Å^−^^3^
3594 reflections	Δρ_min_ = −0.62 e Å^−^^3^
181 parameters	Absolute structure: Flack *x* determined using 1016 quotients [(*I*^+^)-(*I*^-^)]/[(*I*^+^)+(*I*^-^)] (Parsons *et al.*, 2013)
0 restraints	Absolute structure parameter: 0.022 (10)

### 4-Bromo-6-phenyl-6,7-dihydro-5H-furo[2,3-f]isoindol-5-one Special details

**Table:**

Geometry. All esds (except the esd in the dihedral angle between two l.s. planes) are estimated using the full covariance matrix. The cell esds are taken into account individually in the estimation of esds in distances, angles and torsion angles; correlations between esds in cell parameters are only used when they are defined by crystal symmetry. An approximate (isotropic) treatment of cell esds is used for estimating esds involving l.s. planes.

### 4-Bromo-6-phenyl-6,7-dihydro-5H-furo[2,3-f]isoindol-5-one Fractional atomic coordinates and isotropic or equivalent isotropic displacement parameters (Å^2^)

**Table:**

	*x*	*y*	*z*	*U*_iso_*/*U*_eq_	
C2	0.1419 (11)	0.4353 (3)	0.53657 (19)	0.0205 (10)	
H2A	0.039792	0.449845	0.502063	0.025*	
C3	0.2950 (10)	0.3444 (3)	0.54847 (16)	0.0200 (8)	
H3A	0.320895	0.285576	0.524699	0.024*	
C3A	0.4127 (10)	0.3535 (3)	0.60449 (17)	0.0167 (9)	
C4	0.5804 (10)	0.2896 (3)	0.64168 (19)	0.0161 (9)	
C4A	0.6479 (10)	0.3280 (3)	0.69413 (18)	0.0147 (9)	
C5	0.8145 (11)	0.2779 (3)	0.74191 (17)	0.0163 (9)	
C7	0.6555 (10)	0.4511 (3)	0.76800 (17)	0.0150 (9)	
H7A	0.811196	0.511277	0.769853	0.018*	
H7B	0.461702	0.465597	0.791989	0.018*	
C7A	0.5487 (10)	0.4299 (3)	0.70951 (19)	0.0155 (9)	
C8	0.3782 (11)	0.4975 (3)	0.67421 (19)	0.0188 (10)	
H8A	0.311613	0.566361	0.684592	0.023*	
C8A	0.3142 (10)	0.4549 (3)	0.62221 (18)	0.0170 (8)	
C11	0.9486 (9)	0.3387 (3)	0.83835 (17)	0.0149 (8)	
C12	1.1382 (11)	0.2504 (3)	0.8542 (2)	0.0198 (10)	
H12A	1.187944	0.197127	0.827962	0.024*	
C13	1.2511 (13)	0.2415 (3)	0.90762 (18)	0.0220 (9)	
H13A	1.378112	0.181725	0.917973	0.026*	
C14	1.1824 (11)	0.3186 (3)	0.94674 (19)	0.0215 (10)	
H14A	1.259007	0.311386	0.983614	0.026*	
C15	1.0007 (11)	0.4061 (3)	0.9311 (2)	0.0216 (10)	
H15A	0.954266	0.459313	0.957452	0.026*	
C16	0.8850 (10)	0.4174 (3)	0.87735 (19)	0.0178 (9)	
H16A	0.763101	0.478337	0.867127	0.021*	
Br1	0.71800 (10)	0.15364 (3)	0.61842 (2)	0.01845 (12)	
N6	0.8208 (8)	0.3525 (2)	0.78384 (14)	0.0160 (7)	
O1	0.1493 (8)	0.5055 (2)	0.58015 (12)	0.0221 (8)	
O2	0.9264 (8)	0.1878 (2)	0.74491 (13)	0.0206 (7)	

### 4-Bromo-6-phenyl-6,7-dihydro-5H-furo[2,3-f]isoindol-5-one Atomic displacement parameters (Å^2^)

**Table:**

	*U*^11^	*U*^22^	*U*^33^	*U*^12^	*U*^13^	*U*^23^
C2	0.020 (2)	0.026 (2)	0.015 (2)	0.0007 (18)	−0.0004 (19)	−0.0008 (18)
C3	0.019 (2)	0.025 (2)	0.016 (2)	−0.004 (2)	0.0013 (18)	−0.0036 (18)
C3A	0.0174 (18)	0.016 (2)	0.017 (2)	−0.0025 (17)	0.0048 (16)	0.0012 (18)
C4	0.015 (2)	0.010 (2)	0.023 (3)	−0.0015 (15)	0.0071 (18)	−0.0017 (17)
C4A	0.0145 (19)	0.010 (2)	0.020 (2)	−0.0007 (14)	0.0036 (16)	0.0018 (15)
C5	0.018 (2)	0.0134 (18)	0.018 (2)	−0.0019 (16)	0.0033 (19)	0.0004 (16)
C7	0.017 (2)	0.0124 (19)	0.016 (2)	0.0004 (15)	0.0021 (18)	0.0000 (16)
C7A	0.016 (2)	0.012 (2)	0.019 (2)	−0.0004 (16)	0.0037 (18)	−0.0009 (17)
C8	0.017 (2)	0.015 (2)	0.024 (3)	0.0031 (16)	0.0043 (18)	−0.0005 (19)
C8A	0.0164 (19)	0.0164 (18)	0.018 (2)	0.0002 (15)	0.000 (2)	0.0032 (17)
C11	0.0139 (19)	0.014 (2)	0.017 (2)	−0.0021 (17)	0.0039 (16)	0.0023 (18)
C12	0.019 (2)	0.014 (2)	0.027 (3)	−0.0009 (16)	0.000 (2)	0.0022 (18)
C13	0.020 (2)	0.0184 (19)	0.027 (2)	−0.002 (2)	−0.001 (2)	0.0050 (17)
C14	0.022 (2)	0.023 (2)	0.020 (2)	−0.0056 (18)	0.001 (2)	0.0036 (17)
C15	0.017 (2)	0.022 (2)	0.026 (3)	−0.0022 (18)	0.005 (2)	−0.005 (2)
C16	0.0175 (19)	0.015 (2)	0.020 (2)	0.0000 (14)	0.000 (2)	−0.0019 (19)
Br1	0.02005 (19)	0.01300 (17)	0.0223 (2)	0.00054 (17)	0.0027 (2)	−0.00368 (18)
N6	0.0195 (17)	0.0117 (15)	0.0168 (17)	−0.0017 (16)	0.0024 (14)	−0.0014 (15)
O1	0.027 (2)	0.0178 (16)	0.0215 (18)	0.0016 (13)	−0.0013 (14)	0.0030 (13)
O2	0.0301 (18)	0.0098 (14)	0.0217 (19)	0.0055 (12)	−0.0003 (14)	0.0004 (12)

### 4-Bromo-6-phenyl-6,7-dihydro-5H-furo[2,3-f]isoindol-5-one Geometric parameters (Å, º)

**Table:**

C2—C3	1.342 (6)	C7A—C8	1.393 (6)
C2—O1	1.384 (5)	C8—C8A	1.396 (6)
C2—H2A	0.9500	C8—H8A	0.9500
C3—C3A	1.443 (5)	C8A—O1	1.376 (5)
C3—H3A	0.9500	C11—C16	1.401 (6)
C3A—C4	1.389 (6)	C11—C12	1.411 (6)
C3A—C8A	1.416 (5)	C11—N6	1.429 (5)
C4—C4A	1.389 (6)	C12—C13	1.378 (6)
C4—Br1	1.902 (4)	C12—H12A	0.9500
C4A—C7A	1.407 (5)	C13—C14	1.392 (6)
C4A—C5	1.482 (6)	C13—H13A	0.9500
C5—O2	1.233 (4)	C14—C15	1.385 (6)
C5—N6	1.391 (5)	C14—H14A	0.9500
C7—N6	1.470 (5)	C15—C16	1.390 (6)
C7—C7A	1.506 (6)	C15—H15A	0.9500
C7—H7A	0.9900	C16—H16A	0.9500
C7—H7B	0.9900		
			
C3—C2—O1	112.5 (4)	C7A—C8—H8A	123.0
C3—C2—H2A	123.8	C8A—C8—H8A	123.0
O1—C2—H2A	123.8	O1—C8A—C8	125.2 (4)
C2—C3—C3A	106.5 (4)	O1—C8A—C3A	109.7 (4)
C2—C3—H3A	126.8	C8—C8A—C3A	125.2 (4)
C3A—C3—H3A	126.8	C16—C11—C12	119.0 (4)
C4—C3A—C8A	118.2 (4)	C16—C11—N6	118.1 (4)
C4—C3A—C3	136.3 (4)	C12—C11—N6	122.9 (4)
C8A—C3A—C3	105.5 (4)	C13—C12—C11	119.9 (4)
C4A—C4—C3A	118.9 (4)	C13—C12—H12A	120.0
C4A—C4—Br1	122.3 (3)	C11—C12—H12A	120.0
C3A—C4—Br1	118.8 (3)	C12—C13—C14	121.1 (4)
C4—C4A—C7A	120.7 (4)	C12—C13—H13A	119.4
C4—C4A—C5	130.8 (4)	C14—C13—H13A	119.4
C7A—C4A—C5	108.5 (4)	C15—C14—C13	119.0 (4)
O2—C5—N6	125.8 (4)	C15—C14—H14A	120.5
O2—C5—C4A	127.6 (4)	C13—C14—H14A	120.5
N6—C5—C4A	106.6 (3)	C14—C15—C16	121.1 (4)
N6—C7—C7A	102.8 (3)	C14—C15—H15A	119.4
N6—C7—H7A	111.2	C16—C15—H15A	119.4
C7A—C7—H7A	111.2	C15—C16—C11	119.8 (4)
N6—C7—H7B	111.2	C15—C16—H16A	120.1
C7A—C7—H7B	111.2	C11—C16—H16A	120.1
H7A—C7—H7B	109.1	C5—N6—C11	126.7 (3)
C8—C7A—C4A	123.1 (4)	C5—N6—C7	112.5 (3)
C8—C7A—C7	127.5 (4)	C11—N6—C7	120.6 (3)
C4A—C7A—C7	109.5 (4)	C8A—O1—C2	105.9 (3)
C7A—C8—C8A	113.9 (4)		
			
O1—C2—C3—C3A	0.5 (5)	C4—C3A—C8A—O1	−179.3 (3)
C2—C3—C3A—C4	178.6 (5)	C3—C3A—C8A—O1	−0.4 (4)
C2—C3—C3A—C8A	0.0 (4)	C4—C3A—C8A—C8	1.4 (6)
C8A—C3A—C4—C4A	−0.8 (6)	C3—C3A—C8A—C8	−179.7 (4)
C3—C3A—C4—C4A	−179.3 (4)	C16—C11—C12—C13	1.5 (6)
C8A—C3A—C4—Br1	−179.5 (3)	N6—C11—C12—C13	−179.4 (4)
C3—C3A—C4—Br1	2.1 (7)	C11—C12—C13—C14	−0.2 (7)
C3A—C4—C4A—C7A	−0.1 (6)	C12—C13—C14—C15	−0.8 (7)
Br1—C4—C4A—C7A	178.6 (3)	C13—C14—C15—C16	0.5 (7)
C3A—C4—C4A—C5	178.8 (4)	C14—C15—C16—C11	0.8 (6)
Br1—C4—C4A—C5	−2.6 (6)	C12—C11—C16—C15	−1.8 (6)
C4—C4A—C5—O2	−1.2 (8)	N6—C11—C16—C15	179.0 (4)
C7A—C4A—C5—O2	177.7 (4)	O2—C5—N6—C11	−1.8 (7)
C4—C4A—C5—N6	179.0 (4)	C4A—C5—N6—C11	178.0 (3)
C7A—C4A—C5—N6	−2.1 (5)	O2—C5—N6—C7	−177.8 (4)
C4—C4A—C7A—C8	0.4 (6)	C4A—C5—N6—C7	2.0 (5)
C5—C4A—C7A—C8	−178.6 (4)	C16—C11—N6—C5	−170.0 (4)
C4—C4A—C7A—C7	−179.5 (4)	C12—C11—N6—C5	10.9 (6)
C5—C4A—C7A—C7	1.4 (5)	C16—C11—N6—C7	5.7 (5)
N6—C7—C7A—C8	179.8 (4)	C12—C11—N6—C7	−173.4 (4)
N6—C7—C7A—C4A	−0.2 (4)	C7A—C7—N6—C5	−1.2 (4)
C4A—C7A—C8—C8A	0.1 (6)	C7A—C7—N6—C11	−177.4 (3)
C7—C7A—C8—C8A	−179.9 (4)	C8—C8A—O1—C2	179.9 (4)
C7A—C8—C8A—O1	179.8 (4)	C3A—C8A—O1—C2	0.7 (4)
C7A—C8—C8A—C3A	−1.0 (6)	C3—C2—O1—C8A	−0.7 (5)

### 4-Bromo-6-phenyl-6,7-dihydro-5H-furo[2,3-f]isoindol-5-one Hydrogen-bond geometry (Å, º)

**Table:**

*D*—H···*A*	*D*—H	H···*A*	*D*···*A*	*D*—H···*A*
C7—H7*A*···O2^i^	0.99	2.51	3.463 (5)	162
C8—H8*A*···O2^ii^	0.95	2.49	3.348 (5)	149
C12—H12*A*···O2	0.95	2.27	2.894 (6)	122
C16—H16*A*···Br1^ii^	0.95	2.97	3.864 (4)	157

Symmetry codes: (i) −*x*+2, *y*+1/2, −*z*+3/2; (ii) −*x*+1, *y*+1/2, −*z*+3/2.

## References

[bb1] Alekseeva, K. A., Fedoseeva, M. A., Bakhanovich, O. V., Khrustalev, V. N., Potkin, V. I., Zhou, H., Nikitina, E. V., Zaytsev, V. P. & Zubkov, F. I. (2024). *J. Org. Chem.* **89**, 3065–3071. 10.1021/acs.joc.3c02533 38359403

[bb2] Alekseeva, K. A., Kvyatkovskaya, E. A., Nikitina, E. V., Zaytsev, V. P., Eroshkina, S. M., Shikhaliev, K. S., Truong, H. H., Khrustalev, V. N. & Zubkov, F. I. (2020). *Synlett* **31**, 255–260.

[bb3] Alekseeva, K. A., Nadirova, M. A., Zaytsev, V. P., Nikitina, E. V., Grigoriev, M. S., Novikov, A. P., Kolesnik, I. A., Mayer, B., Müller, T. J. J. & Zubkov, F. I. (2023). *J. Org. Chem.* **88**, 15029–15040. 10.1021/acs.joc.3c01476 37870950

[bb4] Apponyi, M. A., Bowie, J. H., Skelton, B. W. & White, A. H. (2002). *Aust. J. Chem.* **55**, 343–348.

[bb5] Bernstein, J., Davis, R. E., Shimoni, L. & Chang, N.-L. (1995). *Angew. Chem. Int. Ed. Engl.* **34**, 1555–1573.

[bb6] Bruker (2007). *APEX2* and *SAINT*. Bruker AXS Inc., Madison, Wisconsin, USA.

[bb7] Deng, M., Yao, Y., Li, X., Li, N., Zhang, X. & Liang, G. (2019). *Org. Lett.* **21**, 3290–3294. 10.1021/acs.orglett.9b01021 31008614

[bb8] Farrugia, L. J. (2012). *J. Appl. Cryst.* **45**, 849–854.

[bb9] Groom, C. R., Bruno, I. J., Lightfoot, M. P. & Ward, S. C. (2016). *Acta Cryst.* B**72**, 171–179. 10.1107/S2052520616003954 PMC4822653 27048719

[bb10] He, Y., Li, X.-M., Hong, T.-Y. & Yang, J.-K. (2022). *CSD Communication*.

[bb11] Horak, Y. I., Lytvyn, R. Z., Zubkov, F. I., Nikitina, E. V., Homza, Y. V., Lis, T., Kinzhybalo, V. & Obushak, M. D. (2013). *Acta Cryst.* E**69**, o273–o274. 10.1107/S160053681300144X PMC3569801 23424547

[bb12] Krause, L., Herbst-Irmer, R., Sheldrick, G. M. & Stalke, D. (2015). *J. Appl. Cryst.* **48**, 3–10. 10.1107/S1600576714022985 PMC4453166 26089746

[bb13] Krishna, G., Grudinin, D. G., Nikitina, E. V. & Zubkov, F. I. (2022). *Synthesis* **54**, 797–863.

[bb14] Ou-Ichen, Z., Boussetta, A., Ouchetto, K., Hafid, A., Khouili, M. & Ouchetto, H. (2024). *J. Iran. Chem. Soc.* **21**, 1453–1493.

[bb15] Parsons, S., Flack, H. D. & Wagner, T. (2013). *Acta Cryst.* B**69**, 249–259. 10.1107/S2052519213010014 PMC3661305 23719469

[bb16] Sheldrick, G. M. (2008). *Acta Cryst.* A**64**, 112–122. 10.1107/S0108767307043930 18156677

[bb17] Sheldrick, G. M. (2015). *Acta Cryst.* C**71**, 3–8.

[bb18] Spackman, P. R., Turner, M. J., McKinnon, J. J., Wolff, S. K., Grimwood, D. J., Jayatilaka, D. & Spackman, M. A. (2021). *J. Appl. Cryst.* **54**, 1006–1011. 10.1107/S1600576721002910 PMC8202033 34188619

[bb19] Speck, K. & Magauer, T. (2013). *Beilstein J. Org. Chem.* **9**, 2048–2078. 10.3762/bjoc.9.243 PMC3817534 24204418

[bb20] Spek, A. L. (2020). *Acta Cryst.* E**76**, 1–11. 10.1107/S2056989019016244 PMC6944088 31921444

[bb21] Venkatesan, P., Thamotharan, S., Ilangovan, A., Liang, H. & Sundius, T. (2016). *Spectrochim. Acta A Mol. Biomol. Spectrosc.* **153**, 625–636. 10.1016/j.saa.2015.09.002 26452098

[bb22] Voronov, A. A., Alekseeva, K. A., Ryzhkova, E. A., Zarubaev, V. V., Galochkina, A. V., Zaytsev, V. P., Majik, M. S., Tilve, S. G., Gurbanov, A. V. & Zubkov, F. I. (2018). *Tetrahedron Lett.* **59**, 1108–1111.

[bb23] Weintraub, R. A. & Wang, X. (2023). *Synthesis* **55**, 519–546.

[bb24] Zaytsev, V. P., Chervyakova, L. V., Sorokina, E. A., Vasilyev, K. A., Çelikesir, S. T., Akkurt, M. & Bhattarai, A. (2021). *Acta Cryst.* E**77**, 86–90. 10.1107/S2056989020016801 PMC7869551 33614131

[bb25] Zaytsev, V. P., Lovtsevich, L. V., Pokazeev, K. M., Sorokina, E. A., Dorovatovskii, P. V., Khrustalev, V. N., Romanycheva, A. A., Shetnev, A. A., Volobueva, A. S., Esaulkova, I. L., Slita, A. V., Zarubaev, V. V. & Zubkov, F. I. (2023). *Tetrahedron* **131**, 133205.

[bb26] Zhou, L., Zhang, M., Li, W. & Zhang, J. (2014). *Angew. Chem. Int. Ed.* **53**, 6542–6545. 10.1002/anie.201403709 24838396

[bb27] Zubkov, F. I., Zaytsev, V. P., Mertsalov, D. F., Nikitina, E. V., Horak, Y. I., Lytvyn, R. Z., Homza, Y. V., Obushak, M. D., Dorovatovskii, P. V., Khrustalev, V. N. & Varlamov, A. V. (2016). *Tetrahedron* **72**, 2239–2253.

